# The Role of the Neighborhood Social Environment on Adulthood Depression: Insights from Midlife in the United States III

**DOI:** 10.1007/s10597-025-01500-w

**Published:** 2025-08-07

**Authors:** Breanna J. Rogers, Yangyang Deng, Mohammad Moniruzzaman, Kosuke Tamura

**Affiliations:** Socio-Spatial Determinants of Health (SSDH) Laboratory, Population and Community Health Sciences Branch, Division of Intramural Research, National Institute on Minority Health and Health Disparities, National Institutes of Health, Bethesda, 20892 MD USA

**Keywords:** Depressive symptoms, Cohesive community, Safe environment, Elderly

## Abstract

Major depressive disorder (MDD) is a serious public health concern in the United States. Prior research has shown that neighborhood characteristics serve as protective factors against depression in adolescents. Few studies have examined the association between perceived neighborhood characteristics and depression during middle and older adulthood. We examined the association between each perceived neighborhood social environment (i.e., social cohesion and safety) and the presence of MDD among Midlife in the United States III (MIDUS) participants (*n* = 2,435, mean age = 63.6 years, Female = 54.4%). Moreover, we investigated whether these associations were moderated by sex and income, separately. All models were adjusted for demographic variables. Overall, perceived neighborhood social cohesion and safety were negatively associated with the presence of MDD. The associations varied when analyses were stratified by sex and income. Findings offer support for the perceived neighborhood social environments as protective factors against depression during middle and older adulthood.

**Supplementary Information:**

The online version contains supplementary material available at 10.1007/s10597-025-01500-w.

## Introduction

Major depressive disorder (MDD) is one of the most serious and widespread public health concerns in the United States (U.S.). Approximately 18.5% U.S. adults reported a diagnosis of MDD in 2020 (Lee et al., 2023; Marr et al., 2022). With an estimated $362 billion in 2020, the direct and indirect cost of depression in the U.S. has substantially risen (Greenberg et al., 2021). Existing evidence suggests that the odds of experiencing lifetime depression are increased for specific sex, racial and/or ethnic, and socioeconomic groups (Hasin et al., 2018; Lee et al., 2023; Vyas et al., 2020). The estimated prevalence of lifetime depression in 2020 was 23.4% among women compared to 13.1% among men (Lee et al., 2023). Among racial and/or ethnic groups, lower prevalence rates of lifetime depression were observed for Asian, Black, Native Hawaiians, and Hispanic or Latino adults (7.6%, 16.1%,15.1%, and 14.6%, respectively) compared to non-Hispanic (NH) White adults (Lee et al., 2023). Conversely, American Indian/Alaskan Native and non-Hispanic multiracial adults had higher lifetime prevalence rates of lifetime depression (23.3% and 28.5%, respectively) (Lee et al., 2023). The prevalence of depression is highest for individuals below the federal poverty threshold compared to individuals above the federal poverty threshold (Brody et al., 2018; Zare et al., 2022). According to the National Center for Health Statistics, approximately 16% of adults living under the federal poverty line experienced depression compared to 3.5% of adults living at or above 400% of the federal poverty line between 2013 and 2016 (Brody et al., 2018).

Many psychological and social determinants have been shown to decrease depressive symptoms in adults. For instance, studies investigating the social determinants of depression often focus on factors such as sex, racial and/or ethnic groups, employment, and the built environment (Remes et al., 2021). In particular, there is a growing body of research investigating the role of the perceived neighborhood social environment (PNSE) in adulthood depression. Similar to objectively measured neighborhood characteristics (e.g., housing quality, air pollution, deprivation) (Cutrona et al., 2006; Neally et al., 2022), individual perceptions of neighborhood characteristics are important to investigate because PNSE factors are related to key multiple processes underlying depression, such as psychological distress and emotional reactivity (Hackman et al., 2019). Furthermore, previous studies indicated that favorable perceived neighborhood social environment characteristics (e.g., less social disorder, more safety) could serve as a protective factor against depression (Andrews et al., 2021; Cutrona et al., 2006).

One particular PNSE (i.e., neighborhood social cohesion)—defined as interconnectedness and solidarity within a community—has been demonstrated to reduce depressive symptoms among young adults and older adults (Breedvelt et al., 2022; Ruiz et al., 2019). Longitudinal studies have provided evidence for a negative association between perceptions of neighborhood social cohesion and depressive symptoms, particularly among older adults. Compared to those with high perceived neighborhood social cohesion, adults with low perceived neighborhood social cohesion experienced an increase in depressive symptoms approximately 15 years earlier (Ruiz et al., 2019). In addition, neighborhood crime and violence were positively associated with depressive symptoms among adults (Baranyi et al., 2021; Tamura et al., 2022; Wilson-Genderson & Pruchno, 2013). For example, among older adults aged 50 to 74, individuals reporting lower neighborhood safety experienced higher levels of depressive symptoms (Wilson-Genderson & Pruchno, 2013). However, it is unknown if this inverse association between perceived neighborhood safety and depression is generalizable to middle-aged adults across the U.S.

To date, few studies have investigated the moderating effect of sex and income on the associations between perceived neighborhood social cohesion and neighborhood safety with depression among middle-to-older aged adults using nationally representative data. A recent study has found support for sex as a moderator for these associations in teens, more pronounced in females (Kulchar et al., 2024). Specifically, perceived neighborhood social cohesion and safety were negatively associated with depressive symptoms for both sexes (Kulchar et al., 2024). Another study among adults also indicated that perceived neighborhood social cohesion was negatively related to depressive symptoms in females, but more pronounced in males (Bassett & Moore, 2013). These inconsistent differences by sex in such associations require further investigation. Furthermore, there have been no studies to date that have examined the moderating role of household income on the associations of social cohesion and safety with depression among middle to older-aged U.S. adults.

The primary aim of this study was to examine the association between each PNSE (i.e., social cohesion and safety) and the presence of MDD among middle-to-older aged adults. The secondary aim was to investigate whether sex and household income would moderate this association, separately. Consistent with a prior study conducted among adolescents (Kulchar et al., 2024) and adults (Bassett & Moore, 2013), we hypothesized that perceived neighborhood social cohesion and neighborhood safety would be negatively associated with the presence of MDD. Furthermore, we hypothesized that sex and income would moderate the associations of perceived neighborhood social cohesion and safety with the presence of MDD.

## Methods

### Participants

Midlife in the United States (MIDUS) is a national longitudinal survey that has sought to investigate physical, psychological, and social well-being throughout adulthood. MIDUS collected survey responses from over 7,000 adults aged 25 to 74 during the first wave (MIDUS I) in 1995 (Delaney, 2014; Radler, 2014). Since then, there have been two additional waves of data collection: MIDUS II conducted from 2004 to 2006 and MIDUS III conducted from 2013 to 2014. This cross-sectional and secondary analysis used data from MIDUS III (*n* = 3,294). Respondents were excluded due to missing: (a) demographic information such as race, income, and marital status (*n* = 806) and (b) neighborhood-level information regarding social cohesion and safety (*n* = 53). This resulted in the final sample of 2,435 respondents. MIDUS III received ethical approval for the study protocol from the Institutional Review Board at the University of Wisconsin-Madison. The National Institutes of Health Institutional Review Board did not deem this analysis human subjects research, as this study used pre-existing, publicly available data.

### Measures

#### Depressive Symptoms

Respondents answered the World Health Organization Composite International Diagnostic Interview Short Form (Gigantesco & Morosini, 2008; Kessler et al., 1998). Items were consistent with criteria from the Diagnostic and Statistical Manual of Mental Disorders 5 (DSM-5) for MDD (i.e., anhedonia, constant fatigue, loss of appetite, lethargy, trouble concentrating, negative affect, and recurring thoughts of death within a two-week period) American Psychiatric Association, 2013). Response options ranged from 0 (no presence of MDD) to 7 (higher scores indicate major depressive symptoms). A dichotomous MDD outcome was created to indicate whether a participant did (presence: range ≥ 4 symptoms) or did not (no presence: range < 4 symptoms) have MDD (Jenkins et al., 2024; Ryff et al., 2019).

#### Perceived Neighborhood Social Environmental (PNSE) Factors

Respondents rated whether they agreed with the following two statements about their neighborhood social cohesion: “I can call neighbor for help if needed/People in my neighborhood trust each other.” Responses were rated on a scale of 1 (“A Lot”) to 4 (“Not at All”). Both items were reversed-scored and averaged, with higher average scores indicating higher neighborhood social cohesion (Carbone, 2020; Keyes, 1998; Robinette et al., 2013; Tamura et al., 2025).

Respondents rated whether they agreed with the following two statements about their neighborhood’s safety: “I feel safe being out alone in my neighborhood during the daytime/I feel safe being out alone in my neighborhood during the night.” Responses were rated on a scale of 1 (“A Lot”) to 4 (“Not at All”). Both items were reverse-scored and averaged, with higher average scores indicating higher neighborhood safety (Carbone, 2020; Keyes, 1998; Robinette et al., 2013; Tamura et al., 2025).

#### Covariates

The analyses included potential demographic covariates that could confound the association between PNSE and depressive symptoms, such as age (in years), sex (male/female), race (NH White/Non-White), marital status (married/not married), and total income. Age, sex, race, and household income were not included as covariates in stratified analyses for their respective models. Annual income was expressed in tertiles (T) based on a prior study using MIDUS data (Rodriguez et al., 2023): (1) Low (T1) < $26,000, (2) Medium (T2): $26,000 - $59,917, and (3) High (T3): < $59,917.

### Statistical Analyses

For our descriptive statistics, means and standard deviations (SD) were used for continuous variables, while frequencies with associated percentages were used for categorical variables. Furthermore, our descriptive statistics were stratified by MDD presence (presence/no presence). To address the first objective, multiple linear regression models were used to estimate the association between each separate PNSE and continuous depressive symptoms. Logistic regression models were used to examine the association between each separate PNSE and the presence of depression (negative/positive), expressed as odds ratios (OR) with a 95% confidence interval (95% CI), adjusting for all covariates. The secondary objective aimed to investigate whether sex and income would moderate associations between each PNSE and depressive symptoms, adjusting for all covariates. The significance of the interaction terms between each moderator and PNSE measures was assessed (ESM 4). Unadjusted and age-adjusted models for overall analyses and analyses stratified by sex and income were also presented in the supplementary materials (ESM 1–3). R version 4.2.3 and the R-packages *oddsratio* (Version 2.0.1, Schratz, 2020) and *metan* (Version 1.18.0, Olivoto & Lúcio, 2020) were used to perform all analyses.

## Results

### Descriptive Statistics

The average age of participants was 63.6 (SD ± 11.1, Table 1). More than half of the participants were female (54.4%), and they were predominantly NH White (95.3%), and most were married (68.0%). The median income was $41,200 (IQR = $18,000, $72,500). The average MDD score was 0.58 (SD ± 1.68) with 234 participants reporting the presence of MDD. Mean perceived neighborhood social cohesion and safety were 3.32 (SD ± 0.69) and 3.66 (SD ± 0.54), respectively.

**Table 1:** Participant characteristics stratified by presence of major depressive disorder (MDD; *n* = 2,435)

		MDD (Yes ≥ 4 symptoms vs. No < 4 symptoms)	
	Overall (*n* = 2,435)	Yes (*n* = 234)	No (*n* = 2,201)
Age, mean (SD)	63.6 (11.1)	60.9 (10.5)	63.9 (11.1)
Sex, n (%)			
Male	1,111 (45.6)	71 (30.3)	1,040 (47.3)
Female	1,324 (54.4)	163 (69.7)	1,161 (52.7)
Race, n (%)			
White	2,320 (95.3)	218 (93.2)	2,102 (95.5)
Non-White	115 (4.7)	16 (6.8)	99 (4.5)
Marital Status, n (%)			
Married	1,657 (68.0)	119 (50.9)	1,538 (69.9)
Not Married	778 (32.0)	115 (49.1)	663 (30.1)
Income, median (IQR)	41,200 (18,000, 72,500)	28,875 (10,000, 46,000)	41,700 (20,000, 74,750)
Income tertiles, n (%)			
Low: <$26,000	832 (34.2)	122 (52.1)	710 (32.3)
Medium: $26,000-$59,917	791 (32.5)	73 (31.2)	718 (32.6)
High: >$59,917	812 (33.3)	39 (16.7)	773 (35.1)
Psychosocial Factors			
Depressive Symptoms, mean (SD)	0.58 (1.68)	5.52 (1.06)	0.06 (0.39)
Neighborhood Measures			
Social Cohesion, mean (SD)	3.32 (0.69)	3.09 (0.81)	3.34 (0.67)
Safety, mean (SD)	3.66 (0.54)	3.50 (0.65)	3.67 (0.53)

*SD* Standard deviation; *IQR* Interquartile Range

### Associations Between PNSE and MDD, and Analyses Stratified by Sex and Income

Overall, perceived neighborhood social cohesion and safety were associated with lower likelihood of the presence of MDD (OR = 0.72, 95% CI = 0.60, 0.88; OR = 0.77, 95% CI = 0.61, 0.97, respectively, Table 2). The interaction term between sex and perceived neighborhood social cohesion was marginally significant (*p* =.06, ESM 4 in supplemental file). Neither measure was related to the presence of MDD in males (Table 3). Both perceived neighborhood social cohesion and safety were negatively associated with the presence of MDD among females (OR = 0.72, 95% CI = 0.57, 0.90; OR = 0.77, 95% CI = 0.59, 1.00, respectively). The interaction terms between income levels and both measures were significant (ESM 4). Perceived neighborhood social cohesion and safety were associated with lower likelihood of the presence of MDD among only low-income households (OR = 0.65, 95% CI = 0.50, 0.85; OR = 0.66, 95% CI = 0.50, 0.88, respectively, Table 4). Neither perceived neighborhood social cohesion nor safety was associated with the likelihood of MDD for medium- and high-income households.

**Table 2:** Overall associations between PNSEs^a^ and MDD^b^ (*n* = 2,435)

	Presence of MDD (Yes/No [reference])	
PNSE Factors	OR^e^	95% CI
Social Cohesion	0.72***	0.60, 0.88
Safety	0.77*	0.61, 0.97

*p <.05; **p <.01; ***p <.001; Covariates included age, sex, race, marital status, and income;

^a^*PNSE *Perceived Neighborhood Social Environment; ^b^*MDD *Major Depressive Disorder; ^c^*β *Beta; ^d^*CI *Confidence Interval; ^e^*OR* Odds Ratio

**Table 3:** Sex-specific association between PNSEs^a^ and MDD^b^ (*n* = 2,435)

		Presence of MDD (Yes/No [reference])	
		OR ^e^	95% CI
Male	Social Cohesion	0.74	0.53, 1.05
	Safety	0.77	0.50, 1.28
Female	Social Cohesion	0.72**	0.57, 0.90
	Safety	0.77*	0.59, 1.00

*p <.05; **p <.01; ***p <.001; Covariates = age, race, marital status, and income;

^a^*PNSE* Perceived Neighborhood Social Environment; ^b^*MDD* Major Depressive Disorder; ^c^*β* Beta; ^d^*CI* Confidence Interval; ^e^*OR *Odds Ratio

**Table 4:** Income-specific association between PNSEs^a^ and MDD^b^ (*n* = 2,435)

		Presence of MDD (Yes/No [reference])	
		OR ^e^	95% CI
Low (T1): <$26,000 ^f^	Social Cohesion	0.65**	0.50, 0.85
	Safety	0.66**	0.50, 0.88
Medium (T2): $26,000–59,917	Social Cohesion	0.77	0.56, 1.08
	Safety	0.96	0.62, 1.58
High (T3): >$59,917	Social Cohesion	0.93	0.58, 1.57
	Safety	1.21	0.55, 3.16

*p <.05; **p <.01; ***p <.001; Covariates = age, sex, race, and marital status;

^a^*PNSE *Perceived Neighborhood Social Environment; ^b^*MDD *Major Depressive Disorder; ^c^*β *Beta; ^d^*CI *Confidence Interval; ^e^*OR *Odds Ratio; ^f^*T *tertile

## Discussion

The primary aim of this study was to investigate the association between PNSE factors and MDD among middle-aged and older adults using data from MIDUS. The secondary aim of this study was to examine whether sex and income moderated these aforementioned associations. Consistent with our hypothesis and previous studies (Andrews et al., 2021; Choi & Ailshire, 2024; Li et al., 2023; Urzua et al., 2019; Wang et al., 2022), the overall results indicated that greater perceived neighborhood social cohesion and safety were negatively associated with MDD (Muhammad et al., 2021; Wilson-Genderson & Pruchno, 2013). Sex and income-stratified analyses revealed differential effects by sex and income compared with the overall findings.

Consistent with prior research, our analysis indicated that perceived neighborhood social cohesion was negatively associated with MDD among middle-aged and older adults (Miao et al., 2019; Ruiz et al., 2018; Urzua et al., 2019). A longitudinal study focusing on a cohort of older adults from Central and Eastern Europe indicated that perceived neighborhood social cohesion was related to lower risk for experiencing higher depression throughout a 3-year period (Urzua et al., 2019). Similarly, findings from the English Longitudinal Study of Ageing indicated that perceptions of neighborhood social cohesion were negatively associated with depression outcome trajectories across a 12-year period among older adults in the United Kingdom (Ruiz et al., 2018). Findings from our study provide further evidence for perceived neighborhood social cohesion as a protective factor among middle-aged and older adults in the U.S. Similar to prior results among adolescents and young adults, middle-aged and older adults benefit from community environments with higher interconnectedness and trust among neighbors (Andrews et al., 2021; Kulchar et al., 2024).

 This study showed that higher perceived neighborhood safety was negatively associated with MDD, consistent with prior studies (Muhammad et al., 2021; Pearson et al., 2021; Tamura et al., 2020; Wilson-Genderson & Pruchno, 2013). For instance, one study from New Jersey indicated that older adults’ perceiving neighborhood unsafety had higher depressive symptoms (Wilson-Genderson & Pruchno, 2013). Likewise, one study using the California Health Interview Survey data showed that perceived neighborhood safety was related to psychological health among older adults (Choi & Matz-Costa, 2018). These studies have focused on examining this association among older adults in New Jersey and California (Choi & Matz-Costa, 2018; Wilson-Genderson & Pruchno, 2013). The findings from this study suggest that the association between perceived neighborhood safety and depressive symptoms might be applicable to middle-aged and older adults across the U.S.

Similar to prior research investigating the relationship between PNSE factors and depression among adolescents and young adults (Bassett & Moore, 2013; Giurgescu et al., 2015; Kreski et al., 2018; Kulchar et al., 2024), sex-specific associations demonstrate the differential effects by sex in this association for middle-aged and older adults. Our results are somewhat consistent with previous studies (Bassett & Moore, 2013; Kulchar et al., 2024). Our negative association between perceived neighborhood social cohesion and MDD among men and women is consistent with a prior study among Canadian adults (Bassett & Moore, 2013). This prior study found that increased trust in neighbors and better social ties to a neighborhood decreased the likelihood of reporting depression among men and women. Similar to another study among adolescents (Kulchar et al., 2024), the association between perceived neighborhood social cohesion and depressive symptoms was more pronounced among girls than boys. One explanation for this might be that women tend to have larger social networks within their neighborhoods than men (Campbell & Lee, 1992; Taylor et al., 2016). In contrast to prior research conducted among adolescents (Kulchar et al., 2024), the negative association between perceived neighborhood safety and depressive symptoms was only significant among women. A possible explanation for this contrasting finding might be that depression is more common among older women than older men (Hooker et al., 2019). A recent study found that older women screened positive for depression at higher rates compared to their male counterparts. Our sample consisted of more women who had MDD (*n* = 163) than men (*n* = 71). As such, our findings might reflect this phenomenon, which might have led to insignificant results among men. Future research should strive to replicate this study with a larger sample of middle-aged and older adult men who screen positive for MDD.

Novel examinations of the moderating role of household income on the association between perceptions of neighborhood social environment factors and depression show how income disparities might play into these associations. A prior cross-sectional examination of German adults found that lower wealth was associated with higher levels of depression severity during middle and older adulthood (Hajek & Koenig, 2022). In the United States, adults with lower family savings (below $20,000) had a higher likelihood of experiencing severe depressive symptoms (Ettman et al., 2020). Our findings demonstrated that higher levels of perceived neighborhood social cohesion may act as a protective factor against MDD among low-income (T1: <$26,000) and medium income households (T2: $26,000 - $59,917). Our findings also demonstrate that perceived neighborhood safety was negatively associated with MDD among low-income households. Notably, prior research has shown that perceptions of neighborhood social cohesion and safety tend to be lower among households with lower incomes and less amenities (Adamus-Leach et al., 2012; Davis et al., 2023; Loukaitou-Sideris & Eck, 2007; Wilkie et al., 2021). Nevertheless, our findings demonstrated that lower income households may benefit from a socially cohesive and safe neighborhood as these neighborhood conditions were associated with reducing the odds of experiencing depression among these groups. Prompting neighborhood social cohesion and safety in low-income neighborhoods through increasing access to and improving neighborhood amenities (e.g., sidewalks, community centers, parks, recreational programs, and streetlights) might be the first step to reduce MDD.

### Strengths and Limitations

One major strength of this study is that it provides insight into that neighborhood-level factors which are associated with depression during middle and older adulthood in the United States. Second, this study used data from the Midlife in the United States (MIDUS) study which consists of a nationally representative sample of middle-aged and older adults. Third, this study investigated the role of age, sex, and income levels in the association between PNSE and depressive symptoms.

Despite these strengths, there are several limitations to this study that limit the generalizability of the results, but they offer pathways for future investigations and improvements. One such limitation is the age of the data. MIDUS III was collected between 2013 and 2014. Though the most recent data available with neighborhood variables included, it is possible that results might not generalize to older adults today. Future research should strive to use more recent data—especially data collected during or after the COVID-19 pandemic—to assess if the results remain consistent. Despite this limitation, the results offer retrospective insight into the association between PNSE and depression among middle-aged to older adults (Ketchen et al., 2023). Although MIDUS is a nationally representative study, it lacks racial and/or ethnic diversity. The sample of non-White individuals (*n* = 118) was also limited. Since this study was cross-sectional, we cannot predict how these associations might change over time. Future research should seek to use a longitudinal design to understand the temporality of the associations. Another limitation is the potential for a same-source bias (Chum et al., 2019). Further research is warranted to investigate the potential for a bidirectional relationship between PNSE factors and MDD among U.S. adults. We excluded 829 participants from the analysis of 3,294 due to missing data on race, marital status, income, and PNSE factors, which could bias the results. Overall, we did not observe substantial differences in the distributions of variables between the excluded and the final analytic samples, except for racial composition. Therefore, the risk of selection bias is likely minimal.

## Conclusion

Higher levels of perceived neighborhood social cohesion were associated with lower severity of depressive symptoms and a reduction in the odds of experiencing depression. Perceived neighborhood safety was associated with depression severity but not with the likelihood of experiencing depression. Our study contributes novel findings through examining the moderating role of age, sex, and household income on these associations. The negative association between perceived neighborhood social cohesion and depression was consistent among age groups, sex, and household income. Findings provide insights into the importance of the perceived neighborhood social environment on depression through middle and older adulthood (Ruiz et al., 2018; Tamura et al., 2020). Moreover, findings provide support for the importance of considering neighborhoods and communities as a tool for combating depression among middle-aged and older adults (Cutrona et al., 2006).

## Electronic supplementary material

Below is the link to the electronic supplementary material.

Supplementary Material 1

## Data Availability

Data is available upon request.

## Declarations

### Disclaimer

This research was supported by the Intramural Research Program of the National Institutes of Health (NIH). The contributions of the NIH authors were made as part of their official duties as NIH federal employees, are in compliance with agency policy requirements, and are considered Works of the United States Government. However, the findings and conclusions presented in this paper are those of the authors and do not necessarily reflect the views of the National Institute on Minority Health and Health Disparities, NIH, or the U.S. Department of Health and Human Services.
