# Supplementary material for: The Role of the Neighborhood Social Environment on Adulthood Depression: Insights from Midlife in the United States III
Source: Community Ment Health J. 2025 Aug 7;62(1):37–44. doi: 10.1007/s10597-025-01500-w (PMC12789143; doi:10.1007/s10597-025-01500-w)
Supplement: Supplementary file 1 — Supplementary Material 1 [file 10597_2025_1500_MOESM1_ESM.docx]

ESM 1. Unadjusted and age-adjusted overall associations between PNSEs ^a^

and MDD ^b^ (n = 2,435)

|  | Presence of MDD (Yes/No [reference]) | |
| --- | --- | --- |
|  | OR ^e^ | 95% CI |
|  | Unadjusted | |
| Social Cohesion | 0.62*** | 0.52, 0.74 |
| Safety | 0.61*** | 0.50, 0.76 |
|  | Age-adjusted | |
| Social Cohesion | 0.65*** | 0.54, 0.78 |
| Safety | 0.59*** | 0.48, 0.73 |

Notes. *p < .05; **p <.01; ***p<.001; ^a^ PNSE = Perceived Neighborhood Social

Environment; ^b^ MDD = Major Depressive Disorder; ^c^ Binary MDD = Presence of

Major Depressive Disorder vs No Presence of Major Depressive Disorder;

^d^ β = Beta; ^e^ CI = Confidence Interval; ^f^ OR = Odds Ratio

ESM 2. Unadjusted and age-adjusted sex-specific associations between PNSEs ^a^ and

MDD ^b^ (n = 2,435)

|  |  | Presence of MDD (Yes/No [reference]) | |
| --- | --- | --- | --- |
|  |  | OR ^e^ | 95% CI |
|  |  | Unadjusted | |
| Male | Social Cohesion | 0.61** | 0.44, 0.84 |
|  | Safety | 0.69 | 0.46, 1.12 |
| Female | Social Cohesion | 0.61*** | 0.49, 0.76 |
|  | Safety | 0.67** | 0.52, 0.86 |
|  |  | Age-adjusted | |
| Male | Social Cohesion | 0.64** | 0.47, 0.89 |
|  | Safety | 0.68 | 0.45, 1.09 |
| Female | Social Cohesion | 0.63*** | 0.51, 0.78 |
|  | Safety | 0.64*** | 0.50, 0.83 |

Notes. *p < .05; **p <.01; ***p<.001; ^a^ PNSE = Perceived Neighborhood Social Environment;

^b^ MDD = Major Depressive Disorder; ^c^ β = Beta; ^d^ CI = Confidence Interval; ^e^ OR = Odds RatioESM 3. Unadjusted and age-adjusted income-specific associations between PNSEs ^a^ and MDD ^b^ (n = 2,435)

|  |  | Presence of MDD (Yes/No [reference]) | |
| --- | --- | --- | --- |
|  |  | OR ^e^ | 95% CI |
|  |  | Unadjusted | |
| Low (T1):  < $26,000 ^f^ | Social Cohesion | 0.58*** | 0.45, 0.74 |
|  | Safety | 0.65** | 0.50, 0.85 |
| Medium (T2): $26,000 – 59,917 | Social Cohesion | 0.68* | 0.50, 0.94 |
|  | Safety | 0.85 | 0.57, 1.35 |
| High (T3): > $59,917 | Social Cohesion | 0.84 | 0.53, 1.38 |
|  | Safety | 0.89 | 0.43, 2.18 |
|  |  | Age-adjusted | |
| Low (T1):  < $26,000 ^f^ | Social Cohesion | 0.63*** | 0.49, 0.81 |
|  | Safety | 0.64** | 0.49, 0.84 |
| Medium (T2): $26,000 – 59,917 | Social Cohesion | 0.75 | 0.55, 1.03 |
|  | Safety | 0.85 | 0.56, 1.35 |
| High (T3): > $59,917 | Social Cohesion | 0.85 | 0.53, 1.40 |
|  | Safety | 0.88 | 0.42, 2.17 |

Notes. *p < .05; **p <.01; ***p<.001; ^a^ PNSE = Perceived Neighborhood Social Environment; ^b^ MDD = Major Depressive Disorder; ^c^ β = Beta; ^d^ CI = Confidence Interval; ^e^ OR = Odds Ratio; ^f^ T = tertile

ESM 4. Interaction terms between PNSEs ^a^ and demographic variables (n = 2,435)

| Interaction Terms | P-Values |
| --- | --- |
| Cohesion * Sex | 0.06 |
| Cohesion * Income | 0.00 |
| Safety * Sex | 0.23 |
| Safety * Income | 0.02 |

Notes: ^a^ PNSE = Perceived Neighborhood Social Environment
